# Diagnostic value of interleukins for tuberculous pleural effusion: a systematic review and meta-analysis

**DOI:** 10.1186/s12890-017-0530-3

**Published:** 2017-12-08

**Authors:** Ni Zeng, Chun Wan, Jiangyu Qin, Yanqiu Wu, Ting Yang, Yongchun Shen, Fuqiang Wen, Lei Chen

**Affiliations:** Department of Respiratory and Critical Care Medicine, West China Hospital of Sichuan University and Division of Pulmonary Diseases, State Key Laboratory of Biotherapy of China, Chengdu, 610041 China

**Keywords:** Interleukin, Tuberculous pleural effusion, Diagnosis, Meta-analysis

## Abstract

**Background:**

The ability of interleukins (ILs) to differentiate tuberculous pleural effusion from other types of effusion is controversial. The aim of our study was to summarize the evidence for its use of ruling out or in tuberculous pleural effusion.

**Methods:**

Two investigators independently searched PubMed, EMBASE, Web of Knowledge, CNKI, WANFANG, and WEIPU databases to identify studies assessing diagnostic role of ILs for tuberculous pleural effusion published up to January, 2017. Study quality was assessed using Quality Assessment of Diagnostic Accuracy Studies-2. The pooled diagnostic sensitivity and specificity of ILs were calculated by using Review Manager 5.3. Area under the summary receiver operating characteristic curve (AUC) was used to summarize the overall diagnostic performance of individual markers.

**Results:**

Thirty-eight studies met our inclusion criteria. Pooled sensitivity, specificity and AUC for chosen ILs were as follows: IL-2, 0.67,0.76 and 0.86; IL-6, 0.86, 0.84 and 0.90; IL-12, 0.78, 0.83 and 0.86; IL-12p40, 0.82,0.65 and 0.76; IL-18, 0.87, 0.92 and 0.95; IL-27, 0.93, 0.95 and 0.95; and IL-33, 0.84, 0.80 and 0.88.

**Conclusions:**

Some of these ILs may assist in diagnosing tuberculous pleural effusion, though no single IL is likely to show adequate sensitivity or specificity on its own. Further studies on a large scale with better study design should be performed to assess the diagnostic potential of ILs.

**Electronic supplementary material:**

The online version of this article (10.1186/s12890-017-0530-3) contains supplementary material, which is available to authorized users.

## Background

Tuberculosis remains a leading cause of morbidity and mortality, especially in Asia and Africa with high tuberculosis burden. In China, the prevalence of active pulmonary tuberculosis in 2010 among those older than 15 years was 459/100,000, and the prevalence of smear-positive pulmonary tuberculosis was 66/100,000. [1] Up to 30% of patients with tuberculosis have tuberculous pleural effusion (TPE), in which extrapulmonary involvement causes pleural effusions [2]. Properly treating pleural effusions requires determining whether the effusions are TPEs or another type of effusion.

The gold standard for diagnosing TPE is the isolation of *Mycobacterium tuberculosis (M. tuberculosis)* from samples of either pleural effusion or pleural biopsy. This culturing offers 100% diagnostic specificity, but it usually takes several weeks, delaying diagnosis and increasing the risk that patients are lost to follow-up. In addition, pleural biopsy is invasive and technically difficult to some extent, particularly in children, such that success can depend strongly on the individual performing the biopsy. [3] Detecting granulomas in pleural biopsies can diagnose TPE with approximately 95% specificity, [2–4] but the sensitivity of culture- or granuloma-based methods is limited. [5] Although image-guided biopsies and local anesthetic thoracoscopic (LAT) biopsies can highly evaluated the sensitivity compared to blind pleural biopsy, both those techniques are not recommended as the first procedure for patients presenting with pleural effusions. Thus, this highlights the need for alternative less invasive diagnostic strategies.

TPE is largely the result of pathological immune reactions associated with an increase in cytokines, including interleukins (ILs). [6] ILs are secreted proteins that bind to specific receptors and help mediate communication among leukocytes. For example, IL-12 is essential for initially activating inrerferon(INF)-γ-mediated T cell responses to primary *M. tuberculosis* infection. [7, 8] ILs can promote various types of inflammatory responses, playing a role in activation-induced death of skin keratinocytes, mucosal epithelial cells, and T cells. [9] Evidence that pleural levels of some ILs are elevated in patients with TPE has led investigators to explore their potential for differentiating TPE from other types of pleural effusion. Most studies have looked at only one or a few ILs, and some studies looking at the same ILs have arrived at different conclusions. This led us to systematically review the literature and meta-analyze available data to gain a more comprehensive understanding of the potential of ILs for diagnosing TPE.

## Methods

### Search strategy and study selection

The systematic review was conducted following the PRISMA (Preferred Reporting Items for Systematic Reviews and Meta-Analysis) guidelines. [10] Two investigators independently searched PubMed, EMBASE, Web of Knowledge, CNKI, WANFANG, and WEIPU databases to identify studies assessing the role of ILs in diagnosing TPE published up to January, 2017. Before the full search, we performed a preliminary search to decide on the ILs to include in the review. The following search terms were used: “interleukins *or* IL” *and* “IL-2 *or* IL-6 *or* IL-12 *or* IL-12p40 *or* IL-18 *or* IL-27 *or* IL-33” *and* “tuberculosis” *and* “pleural effusion/pleural fluid” *and* “sensitivity or specificity or accuracy”. Reference lists in retrieved studies and review articles were examined manually to identify additional studies.

Two authors (NZ and CW) independently assessed each study for eligibility; disagreements were resolved by consensus. Studies were included if they fulfilled all the following criteria: (1) the work was an original research article published in English or Chinese, (2) human samples were analyzed, (3) standard methods were used to definitively diagnose the type of effusion as TPE or other type, and (4) data sufficient for calculating specificity and sensitivity were reported. Conference proceedings, letters to the editor, and studies including fewer than 10 patients with TPE were excluded.

### Quality assessment and data extraction

The same two authors (NZ and CW) assessed the quality of included studies using the Quality Assessment of Diagnostic Accuracy Studies-2 tool (QUADAS-2). [11] For each criterion, a response of “yes” was assigned if it was fulfilled; “unclear”, if doubt existed whether it was fulfilled; or “no” if it was not fulfilled. The following data were retrieved from each study: authors, country, publication year, population characteristics, testing methods, cut-off value, methodological quality, and 2-by-2 tables showing rates of true positives (TPs), true negatives (TNs), false positives (FPs) and false negative (FNs).

### Statistical analysis

Data were compiled in Excel, then transferred to Review Manager 5.3 (The Cochrane Collaboration, Copenhagen, Denmark) and STATA Version 12.0 (Stata Corp., College Station, TX) for statistical analysis. For each study, sensitivity, specificity, positive likelihood ratio (PLR), negative likelihood ratio (NLR), and diagnostic odds ratio (DOR) were calculated, together with 95% confidence intervals (CIs). A summary ROC (SROC) curve was generated for each IL in each study, [12] from which a single test threshold value was determined and used to calculate sensitivity and specificity. [13] Overall diagnostic performance for that IL was assessed as the area under the SROC curve (AUC).

The Q test and inconsistency index (I^2^) were used to detect potential heterogeneity in the natural logarithm of DOR (lnDOR) meta-analyzed across studies. [14] Presence of implicit cut-off point effects and correlation between sensitivity and specificity were assessed for each IL by calculating the Spearman rank correlation coefficient for each IL. Deeks’ funnel plot and Egger’s test were used to detect publication bias [15]. All statistical tests were two-sided, with *P* < 0.05 taken as the threshold of significance.

## Results

**Fig. 1:**
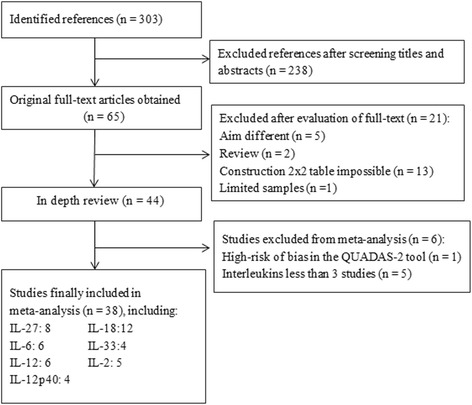
Flow diagram of study selection. QUADAS: Quality Assessment of Diagnostic Accuracy Studies

Our systematic review included 38 studies examining the ability of pleural concentrations of IL-2, IL-6, IL-12, IL-12p40, IL-18, IL-27, and IL-33 to diagnose TPE. [16–53] Other ILs in the Table 1 were excluded for meta-analysis because relevant data were available from fewer than 3 studies [54–58] (Fig. 1). Two authors (NZ and CW) assessed studies for possible overlap in the populations analyzed. Data were pooled from overlapping populations as long as the different studies reported on different ILs or IL combinations. Otherwise, if studies with overlapping populations reported on the same IL or IL combination, only the data from the largest study were used.

**Table 1:** Clinical summary of all studies

Interleukins	Author	Country(incidence)	Year	Cut-off (pg/ml)	Index test	Design
IL-27	Wu et al. [16]	China (high)	2013	900.8	ELISA	P
	Liu et al. [42]	China (high)	2015	1012	ELISA	R
	Luo et al. [27]	China (high)	2015	353.47	ELISA	R
	Skouras et al. [46]	Greece (low)	2015	391	ELISA	NA
	Sun et al. [29]	China (high)	2014	838	ELISA	R
	Valdes et al. [31]	Spain (low)	2014	550	ELISA	P
	Yang et al. [50]	China (high)	2012	1007	ELISA	P
	Niu et al. [38]	China (high)	2012	846	ELISA	R
IL-18	Chen et al. [17]	China (high)	2011	843.7	ELISA	R
	Dai et al. [18]	China (high)	2015	503.58	ELISA	R
	Ding et al. [19]	China (high)	2008	640	ELISA	R
	Hu et al. [21]	China (high)	2009	365	ELISA	R
	Jiang et al. [22]	China (high)	2009	503.88	ELISA	R
	Klimiuk et al. [39]	Poland (low)	2014	327.7	ELISA	P
	Liu et al. [43]	China (high)	2015	438.86	ELISA	R
	Okamoto et al. [44]	Japan (low)	2005	992.7	ELISA	NA
	Wang et al. [47]	China (high)	2008	358	ELISA	R
	Wu et al. [33]	China (high)	2006	150	ELISA	R
	Xiong et al. [34]	China (high)	2007	358	ELISA	R
	Yu et al. [51]	China (high)	2003	150	ELISA	NA
IL-6	Kiropoulos et al. [24]	Greece (low)	2007	17,215	ELISA	P
	Wang et al. [32]	China (high)	2005	1950	ELISA	R
	Wong et al. [48]	China (high)	2003	4000	ELISA	P
	Wu et al. [49]	China (high)	2005	550	ELISA	R
	Zan et al. [35]	China (high)	2014	277	RIA	NA
	Yang et al. [37]	China (high)	2006	220	RIA	R
IL-33	Lee et al. [26]	Korea (low)	2013	10	ELISA	R
	Li et al. [40]	China (high)	2015	68.3	ELISA	R
	Liu et al. [42]	China (high)	2015	19.31	ELISA	R
	Xuan et al. [36]	China (high)	2014	19.86	ELISA	R
IL-12	Chen et al. [17]	China (high)	2011	785.6	ELISA	R
	Gu et al. [20]	China (high)	2002	300	ELISA	NA
	Jiang et al. [23]	China (high)	2010	87.41	ELISA	R
	Okamoto et al. [44]	Japan (low)	2005	129	ELISA	NA
	Tian et al. [28]	China (high)	2004	73.5	ELISA	R
	Zhou et al. [53]	China (high)	2012	90	ELISA	R
IL-2	Liu et al. [43]	China (high)	2015	67.17	ELISA	R
	Liu et al. [42]	China (high)	2015	99.08	ELISA	R
	Ren et al. [25]	China (high)	2014	41.91	ELISA	R
	Wu et al. [49]	China (high)	2005	250	ELISA	R
	Zhang et al. [52]	China (high)	1998	400	RIA	R
IL-12p40	Fernández et al. [41]	Venezuela (low)	2011	89	ELISA	NA
	Klimiuk et al. [39]	Poland (low)	2014	296	ELISA	P
	Tural Önür et al. [45]	Turkey (low)	2015	210	ELISA	NA
	Valdes et al. [30]	Spain (low)	2009	550	ELISA	P
IL-8	Yamada et al. [57]	Japan (low)	2001	228	ELISA	R
	Yang et al. [56]	China (high)	2001	1000	ELISA	R
IL-10	Wu et al. [49]	China (high)	2005	50	ELISA	R
IL-22	Jin et al. [58]	China (high)	2011	49	ELISA	R
	Yuan et al. [55]	China (high)	2014	186.6	ELISA	R
IL-23	Klimiuk et al. [39]	Poland (low)	2014	0.7	ELISA	P
IL-31	Gao et al. [54]	China (high)	2015	67.5	ELISA	R

*Abbreviations*: *IL* interleukin, *ELISA* enzyme-linked immunosorbent assay, *RIA* radioimmunoassay, *NA* not available, *P* prospective, *R* retrospective

### Study characteristics

Table 1 summarizes clinical characteristics of patients in the 38 studies that used for quantitative meta-analysis [16–53]. Average sample size was 98 (range, 43 to 431) for each IL (Table 2). 23 studies stated that the pleural effusion samples were collected before any drug treatment [16–38], while the rest 15 studies didn’t report such information [39–53]. Diagnosis of TPE or other type of pleural effusion was based only on clinical course in 5 studies, [22, 23, 28, 35, 41] i.e. on clinical presentation, pleural fluid analysis, radiology and responsiveness to anti-tuberculosis chemotherapy. Diagnosis was based on bacteriology, histology or both (gold standard) in 11 studies. In the remaining 21 studies, some patients were diagnosed with TPE based on clinical course and others based on the gold standard. One study [51] did not report the diagnostic standard for TPE. All but 3 studies [35, 37, 52] measured IL levels using enzyme-linked immunosorbent assays (ELISA), with the remaining 3 studies using radioimmunoassays.

**Table 2:** Diagnostic performance of interleukins from individual studies

Interleukins	Author	Subjects	TP	FP	FN	TN
IL-27	Wu et al. [16]	81	38	1	2	40
	Liu et al. [42]	147	88	3	7	49
	Luo et al. [27]	62	32	1	2	27
	Skouras et al. [46]	121	8	10	2	101
	Sun et al. [29]	76	38	1	2	35
	Valdes et al. [31]	431	64	54	6	307
	Yang et al. [50]	174	63	1	5	105
	Niu et al. [38]	44	23	1	0	20
IL-18	Chen et al. [17]	64	28	4	6	26
	Dai et al. [18]	52	21	2	2	27
	Ding et al. [19]	72	33	2	1	36
	Hu et al. [21]	102	48	3	4	47
	Jiang et al. [22]	60	26	2	4	28
	Klimiuk et al. [39]	203	27	20	17	139
	Liu et al. [43]	80	36	3	4	37
	Okamoto et al. [44]	43	4	1	7	31
	Wang et al. [47]	44	17	2	2	23
	Wu et al. [33]	48	20	2	4	22
	Xiong et al. [34]	86	41	3	5	37
	Yu et al. [51]	52	30	0	2	20
IL-6	Kiropoulos et al. [24]	97	22	17	3	55
	Wang et al. [32]	71	33	2	1	35
	Wong et al. [48]	66	29	8	3	26
	Wu et al. [49]	109	31	9	25	44
	Zan et al. [35]	56	30	5	12	9
	Yang et al. [37]	54	20	2	2	30
IL-33	Lee et al. [26]	220	47	56	13	104
	Li et al. [40]	87	27	16	5	39
	Liu et al. [42]	147	82	5	13	47
	Xuan et al. [36]	44	20	2	3	19
IL-12	Chen et al. [17]	64	30	5	4	25
	Gu et al. [20]	52	27	5	5	25
	Jiang et al. [23]	60	22	3	8	27
	Okamoto et al. [44]	43	6	1	5	31
	Tian et al. [28]	190	120	17	21	32
	Zhou et al. [53]	73	31	8	14	20
IL-2	Liu et al. [43]	80	25	6	15	34
	Liu et al. [42]	147	53	18	42	34
	Ren et al. [25]	88	39	4	3	42
	Wu et al. [49]	109	34	21	22	32
	Zhang et al. [52]	69	23	6	4	36
IL-12p40	Fernández et al. [41]	60	11	20	9	20
	Klimiuk et al. [39]	203	38	44	6	115
	Tural Önür et al. [45]	120	42	27	10	41
	Valdes et al. [30]	96	36	17	3	40
IL-8	Yamada et al. [57]	70	17	14	4	35
	Yang et al. [56]	64	38	4	2	20
IL-10	Wu et al. [49]	109	46	7	20	36
IL-22	Jin et al. [58]	56	23	1	5	27
	Yuan et al. [55]	87	47	7	5	28
IL-23	Klimiuk et al. [39]	203	13	66	31	93
IL-31	Gao et al. [54]	71	33	0	7	31

*Abbreviations*: *IL* interleukin, *TP* true-positive, *FP* false-positive, *FN* false-negative, *TN* true-negative

### Determination of statistical pooling model

Diagnostic studies are typically meta-analyzed using an SROC-based fixed-effects model, [59] a random-effects model using a bivariate normal approximation, [60] or a hierarchical SROC (HSROC)-based full Bayesian [61] or empirical Bayes method [62]. In our study, lnDOR heterogeneity was statistically significant and associated with high I^2^ values for most ILs (Table 3). These indications of substantial heterogeneity in lnDOR made the use of a SROC-based fixed-effects model inappropriate [63].

**Table 3:** Statistical measures of heterogeneity, cut-off effect, and publication bias for each interleukin

interleukins	I^2^ for heterogeneity in InDOR(%)	Spearman’s coefficient	Egger test *P* value	Deeks test *P* value
IL-27	53.5	−0.467	0.101	0.57
IL-18	61.7	−0.511	0.5	0.73
IL-6	77.8	−0.657	0.532	0.66
IL-33	77.7	−1.00	0.359	0.54
IL-12	34.3	0.493	0.029	0.23
IL-2	88.4	−0.900	0.17	0.18
IL-12p40	83.2	−0.800	0.9	0.34

The possible presence of implicit cut-off point effects was examined for each included IL, using the Spearman rank correlation between sensitivity and specificity (Table 3). A negative correlation was found for most ILs, indicating no detectable implicit cut-point effect. Therefore, we used a random-effects model to estimate the mean sensitivity and specificity and associated CIs.

### Diagnostic accuracy

**Fig. 2:**
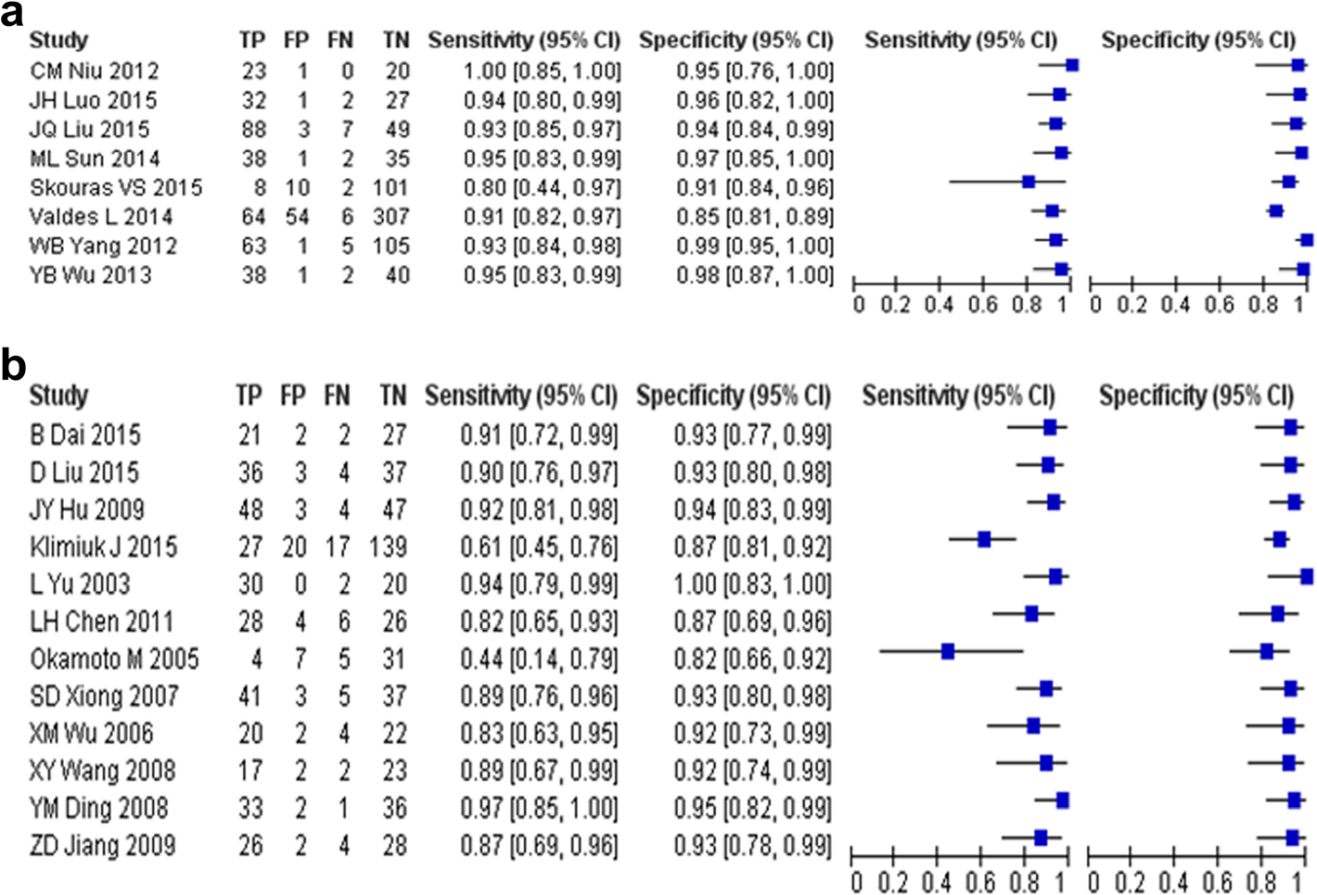
Forest plot of the sensitivities and specificities. **a**. interleukin-27, **b**. interleukin-18. The calculated pooled mean with corresponding confidence interval is also reported

These data were meta-analyzed using a random-effects model (Table 3). Fig. 2 summarizes the sensitivities and specificities for IL-27 and IL-18 reported by each study. (Results for the other ILs are reported in Additional file 1: Figure S1.) Sensitivity of IL-27 ranged from 0.80 to 1.00, and the pooled value was 0.93 (95%CI 0.90–0.95). Sensitivity of IL-18 ranged from 0.44 to 0.97, and the pooled value was 0.87 (95%CI 0.79–0.92). Specificity of IL-27 varied from 0.85 to 0.99, and the pooled value was 0.95 (95%CI 0.90–0.98). Specificity of IL-18 varied from 0.82 to 1.00, and the pooled value was 0.92 (95% CI 0.88–0.95). The pooled parameters for all included ILs are shown in Table 4.

**Table 4:** Pooled means of sensitivity and specificity, diagnostic odds ratio(DOR), area under the curve(AUC), and calculated likelihood ratios for each interleukin

Interleukins	sensitivity(95%CI)	specificity(95%CI)	DOR	AUC	PLR	NLR
IL-27	0.93(0.90–0.95)	0.95(0.90–0.98)	264	0.95	19.5(9.4–40.5)	0.07(0.05–0.11)
IL-18	0.87(0.79–0.92)	0.92 (0.88–0.95)	76	0.95	10.8 (7.2–16.3)	0.14(0.09–0.23)
IL-6	0.86(0.70–0.94)	0.84(0.74–0.90)	30	0.90	5.2(3.0–9.1)	0.17(0.07–0.41)
IL-33	0.84(0.77–0.89)	0.80(0.65–0.89)	20	0.88	4.2(2.21–7.9)	0.20(0.13–0.32)
IL-12	0.78(0.69–0.84)	0.83(0.72–0.91)	17	0.86	4.6(2.8–7.8)	0.27(0.20–0.37)
IL-2	0.67(0.61–0.73)	0.76(0.70–0.82)	11	0.86	3.4(1.7–6.8)	0.36(0.20–0.66)
IL-12p40	0.82(0.66–0.91)	0.65(0.54–0.74)	8	0.76	2.3(1.6–3.4)	0.28(0.13–0.61)

*Abbreviations*: *IL* interleukin, *DOR* diagnostic odds ratio, *AUC* area under the curve, *PLR* positive likelihood ratio, *NLR* negative likelihood ratio

**Fig. 3:**
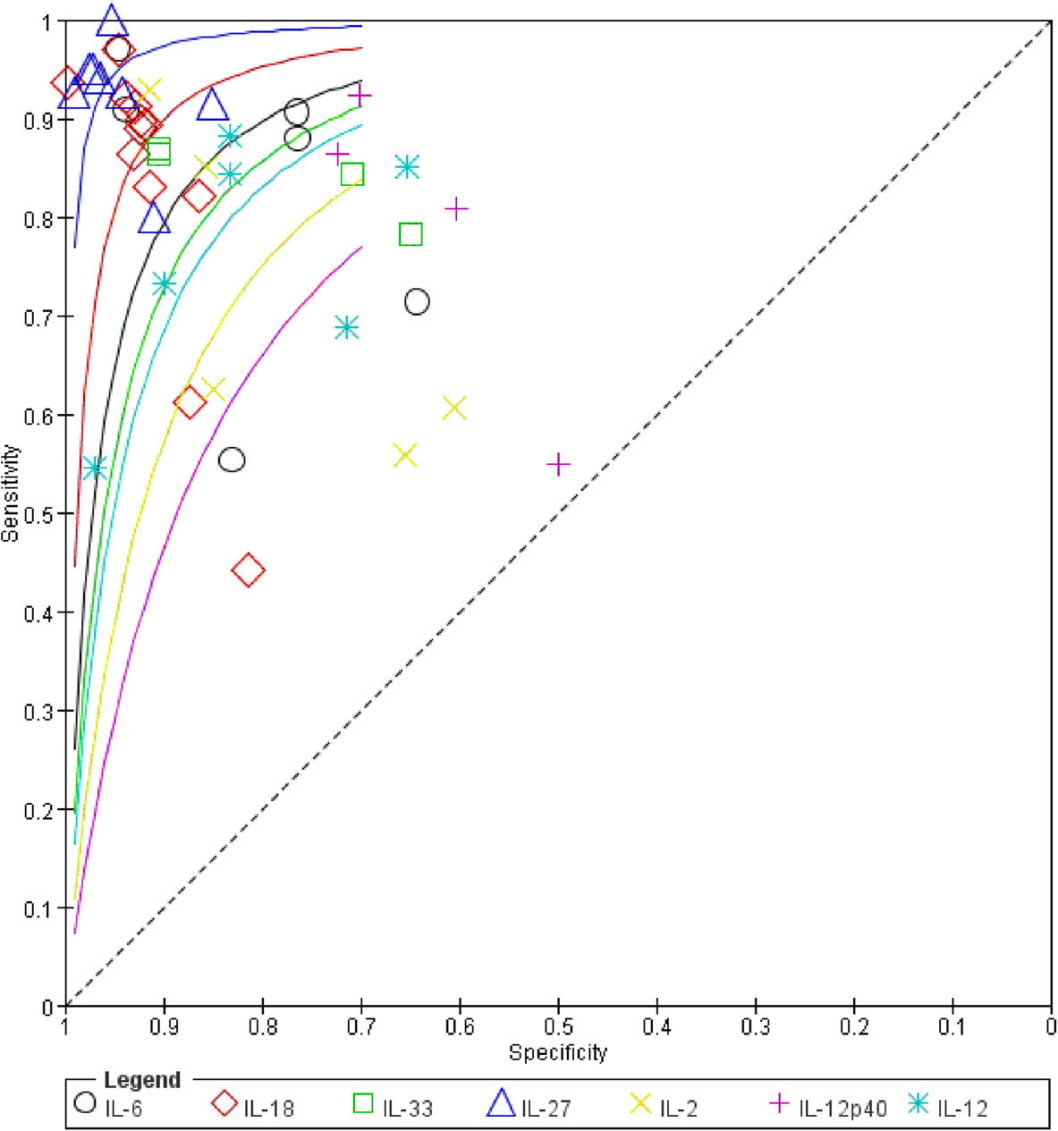
Summary receiver operating characteristic (SROC) curve for all the interleukins included

Unlike a traditional ROC plot, each data point on an SROC curve represents a separate study, allowing the curve to provide an overall assessment of diagnostic performance. Plotting the rate of TP against the rate of FP gave curves showing AUCs of 0.95 for IL-18 and IL-27 (Fig. 3). Among all ILs, IL-27 showed the highest overall accuracy, with a sensitivity of 93% and specificity of 95%.

### Study quality and publication bias

**Fig. 4:**
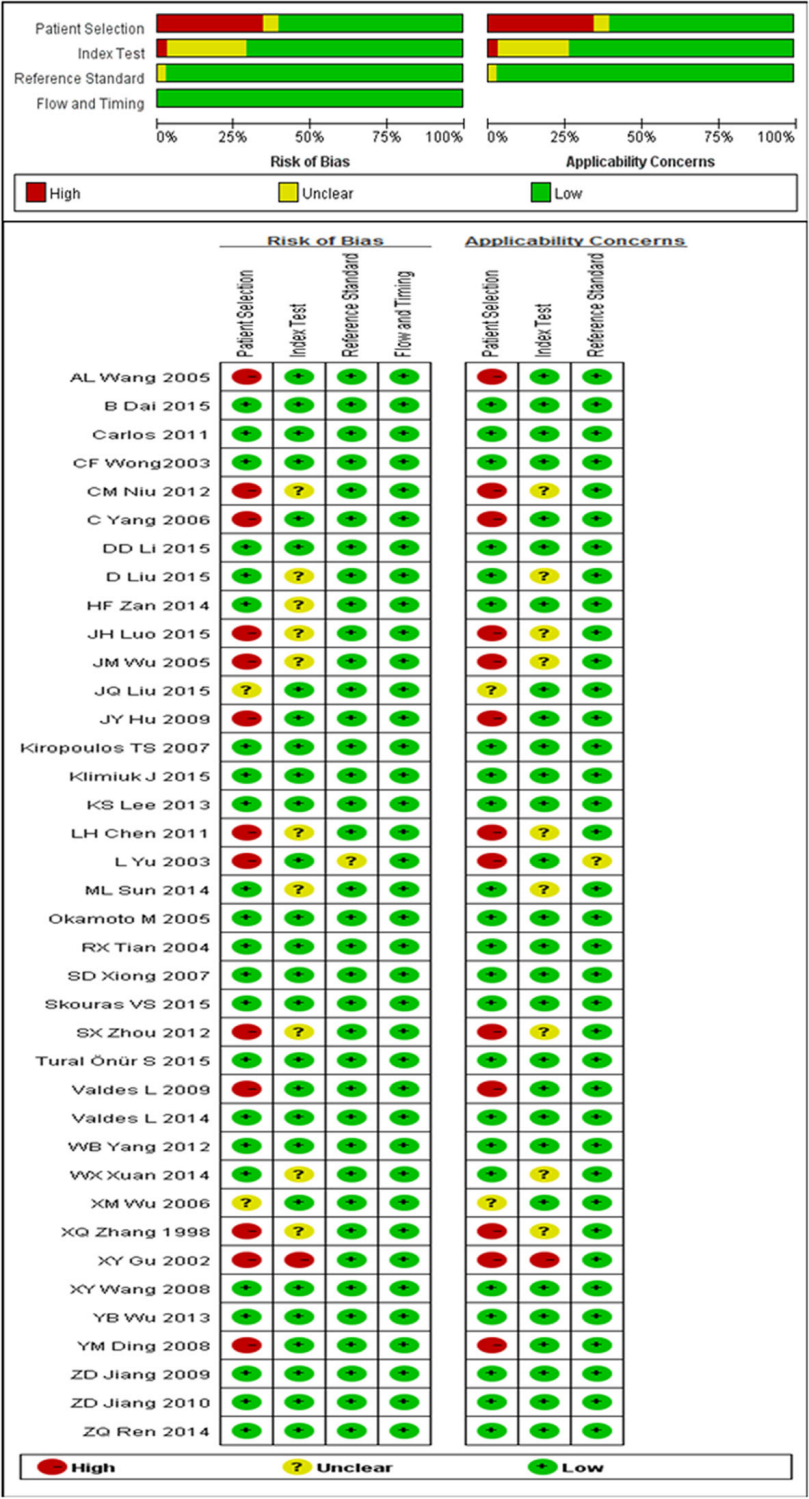
Summary of QUADAS-2 assessments of included studies. QUADAS-2: Quality Assessment of Diagnostic Accuracy Studies-2. Patient Selection: Describe methods of patient selection; Index Text: Describe the index test and how it was conducted and interpreted; Reference Standard: Describe the reference standard and how it was conducted and interpreted; Flow and Timing: Describe any patients who did not receive the index tests or reference standard or who were excluded from the 2 × 2 table, and describe the interval and any interventions between index tests and the reference standard

QUADAS-2 assessment of included studies showed that most studies had low risk of bias (Fig. 4). Both Egger’s and Deeks’ tests suggest no evidence of bias among the studies for any ILs meta-analyzed (Table 3). Funnel plots indicate low risk of publication bias (Additional file 1: Figure S2).

## Discussion

Assaying pleural levels of ILs may be a cost-effective and minimally invasive alternative to traditional tests for differentiating TPE from other types of pleural effusion. Our meta-analysis of the available evidence suggests that IL-27 and IL-18 show relatively high diagnostic accuracy for TPE, while five other well-studied ILs do not (IL-2, IL-12, IL-27, IL-33 and IL-12p40). Even IL-27 and IL-18 do not appear to have adequate diagnostic potential on their own, so they would need to be used in conjunction with other methods or conventional markers.

Our meta-analysis showed that IL-2, despite being centrally involved in the regulation of immune tolerance and activation, [64] is associated with quite low sensitivity and specificity. This may reflect the fact that IL-2 data were available from only 5 studies, all of which were conducted in China. Future work, preferably in Caucasians and other groups of Asians, should investigate the diagnostic potential of this IL.

DOR combines sensitivity and specificity into a single indicator of test performance. [65] Higher DOR indicates better discriminatory test performance. Mean DOR was 64.12 for IL-18 and 227.9 for IL-27, indicating high overall accuracy. Potentially more clinically meaningful than DOR are likelihood ratios. [66] A likelihood ratio > 10 or <0.1 suggests a 10-fold difference between the pre- and post-test probability that a condition is present. Of the ILs meta-analyzed here, only IL-18 and IL-27 had PLRs >10, suggesting that a positive test result for these ILs indicates a relatively high probability of TPE. In addition, IL-27 was associated with an NLR of 0.08, indicating an 8% probability that a negative IL-27 test result is a false negative for TPE. This may be sufficient for ruling out TPE in the clinic.

Pleural levels of a number of biomarkers have been proposed as aids in the diagnosis of TPE, including adenosine deaminase (ADA) and interferon-γ(INF-γ), both of which are present in patients with TPE at significantly higher concentrations than in patients with other types of pleural effusion. The diagnostic performance determined here for IL-18 and IL-27 compares favorably with that of ADA and INF-γ. Meta-analyses [67, 68] indicate that these latter two assays on their own are associated with the following diagnostic indices: sensitivity, 0.89 (95%CI 0.87–0.91) and 0.92 (95%CI 0.90–0.93); specificity, 0.97 (95%CI 0.96–0.98) and 0.90 (95%CI 0.89–0.91); PLR, 23.45 (95%CI 17.31–31.78) and 9.03 (95%CI 7.19–11.35); NLR, 0.11 (95%CI 0.07–0.16) and 0.10 (95%CI 0.07–0.14); and DOR, 272.7 (147.5–504.2) and 110.08 (95%CI 69.96–173.20). Although the available evidence suggests that IL-18 and IL-27 seem to have higher accuracy than ADA, the higher-cost and more complicated determination of IL-27 and IL-18 may limit their practical applicability. [69, 70] In addition, it has been reported that the combination of positive IL-27 with positive ADA values [16, 31, 46], can reach a sensitivity of 100% for the identification of TBP, Our meta-analysis, combined with previous ones, suggests that combining IL-18 and IL-27 with INF-γ and ADA may strengthen TPE diagnosis. We also suggest further studies should be carried out to determine the diagnostic accuracy of IL-27 and IL-18 combination or their combination with ADA or INF-γ.

Our meta-analysis suggests an association between elevated levels of at least certain pleural ILs and TPE. TPE has been characterized as a hypersensitive T cell reaction to mycobacteria or antigens in the pleural space, leading to the accumulation of protein-rich fluid. [6] ILs are divided into different families based on sequence homology, receptor chains or functional properties. IL-18 and IL-33 belong to the IL-1 family, [71] which contains inflammatory mediators playing a major role in early innate immune responses. IL-6, which belongs to a cytokine family of the same name, is a multifunctional, pleiotropic regulator of immune responses, acute-phase responses, hematopoiesis, and inflammation. [72] IL-2, a member of the γ-chain cytokine family, is produced mainly by CD4+ and CD8+ T cells and is essential for Treg cell development. [73] Although both blood and pleural fluid samples can be processed for all ILs, these assays are limited by their inability to differentiate drug resistant TB, consequently, cannot replace appropriate microbiological and molecular investigations. Future work is needed to examine how ILs may affect onset and/or progression of TPE and the probable association between ILs and drug sensitivity of TB.

To ensure reliable results, we meta-analyzed only ILs for which sensitivity and specificity data were available from at least 3 studies. As a result, we did not analyze several ILs for which levels appear to be elevated in tuberculosis [74], including IL-8 [57] and IL-22 [58]. Further work should examine the diagnostic potential of these ILs. In addition, more work should also examine the diagnostic performance of these and other ILs in combination, which we could not do for lack of studies including such combinations.

Our meta-analysis has additional limitations. First, exclusion of conference abstracts, letters to journal editors and unpublished data may have given rise to publication bias, such that our results overestimate actual diagnostic performance. Second, patients were diagnosed with TPE based on both bacteriological and histological assessment in only a few studies; in most studies, patients were diagnosed on the basis of one or the other, alone or in combination with clinical course, and they were diagnosed based solely on clinical course in a few studies. This increases risk of misclassification bias. Third, description of methodology was incomplete in many studies, leading to a QUADAS-2 assessment of “unclear”. In addition, we did not perform meta-regression analysis to determine the source of heterogeneity, because of the limited numbers of the studies included. Our results highlight the need for more rigorous studies of ILs in the diagnosis of TPE. Future work should also examine the diagnostic potential of IL levels in serum, since most studies have focused on pleural levels.

## Conclusion

The available evidence suggests that assaying pleural levels of certain ILs may aid in the diagnosis of TPE when used in combination with other biomarkers and approaches. By confirming such diagnosis, ILs may help avoid the need for more invasive diagnostic procedures.

## Additional files

Additional file 1: Figure S1. Forest plot of the sensitivities and specificities reported by each interleukin. The Forest plots of the sensitivities and specificities reported by A. interleukin-6; B.interleukin-33; C interleukin-12; D interleukin-2; E interleukin-12p40. **Figure S2.** Funnel graph for the assessment of potential publication bias in each interleukin. The Funnel graphs for the assessment of potential publication bias in each interleukin: A for IL-27; B for IL-18;C for IL-6; D for IL-33; E for IL-12; F for IL-2; G for IL-12p40. (DOC 231 kb)

## Abbreviations

ADA: Adenosine deaminase INF-γ Interferon-γ
AUC: The area under the SROC curve
DOR: Diagnostic odds ratio
FN: False negative
FP: False positive
IL: Interleukins
NLR: Negative likelihood ratio
PLR: Positive likelihood ratio
QUADAS-2: Quality Assessment of Diagnostic Accuracy Studies-2
SROC: Summary ROC
TN: True negative
TP: True positive
TPE: Tuberculous pleural effusion
