# Supplementary material for: Diagnostic value of interleukins for tuberculous pleural effusion: a systematic review and meta-analysis
Source: BMC Pulm Med. 2017 Dec 8;17:180. doi: 10.1186/s12890-017-0530-3 (PMC5721598; doi:10.1186/s12890-017-0530-3)

**e-Figure 1.** Forest plot of the sensitivities and specificities reported by each article for ：A. interleukin-6; B.interleukin-33; C interleukin-12; D interleukin-2; E interleukin-12p40

A.


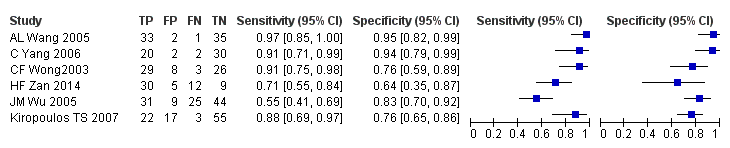


B.


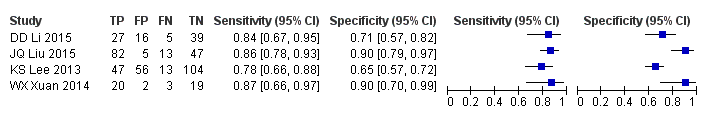


C.


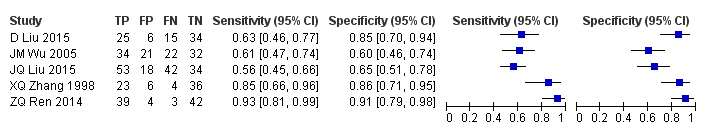


D

.
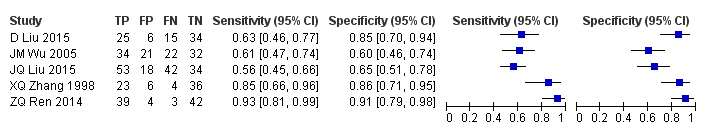


E

.
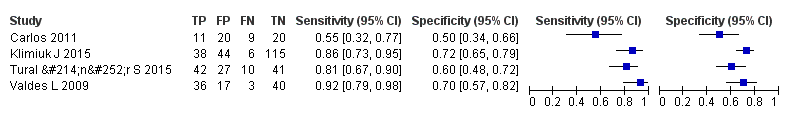


**e-Figure 2:**Funnel graph for the assessment of potential publication bias in each interleukin assays. A for IL-27; B for IL-18;C for IL-6; D for IL-33; E for IL-12; F for IL-2; G for IL-12p40.

A.


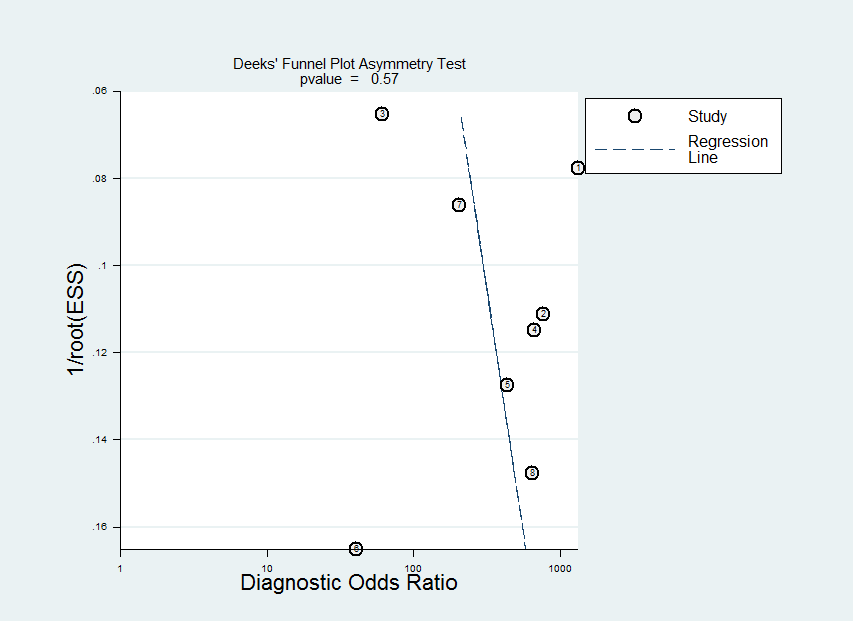


B.


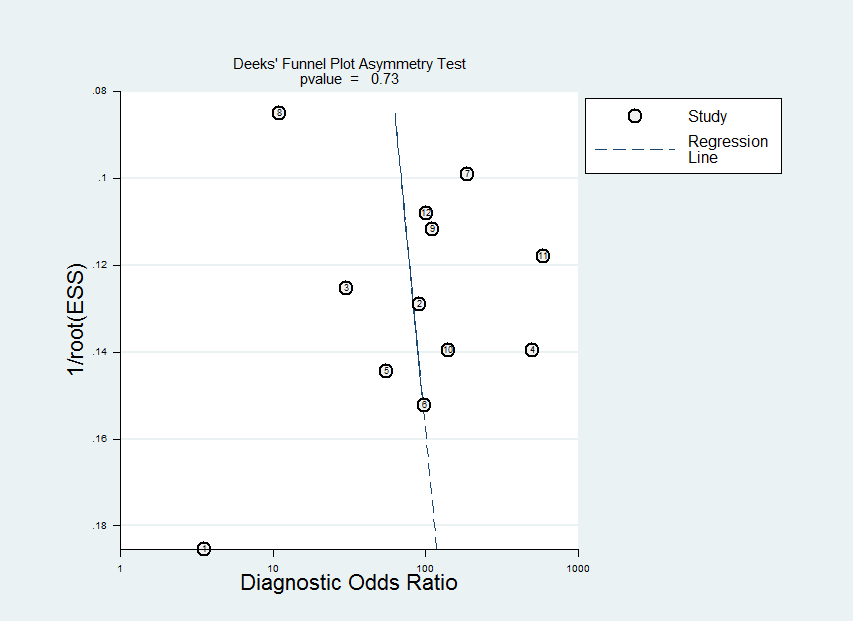


C.


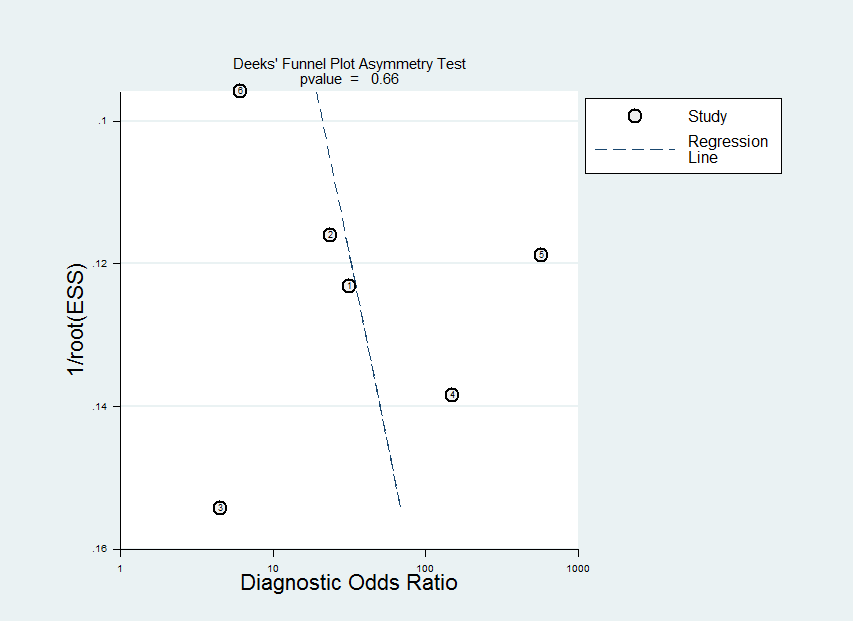


D.


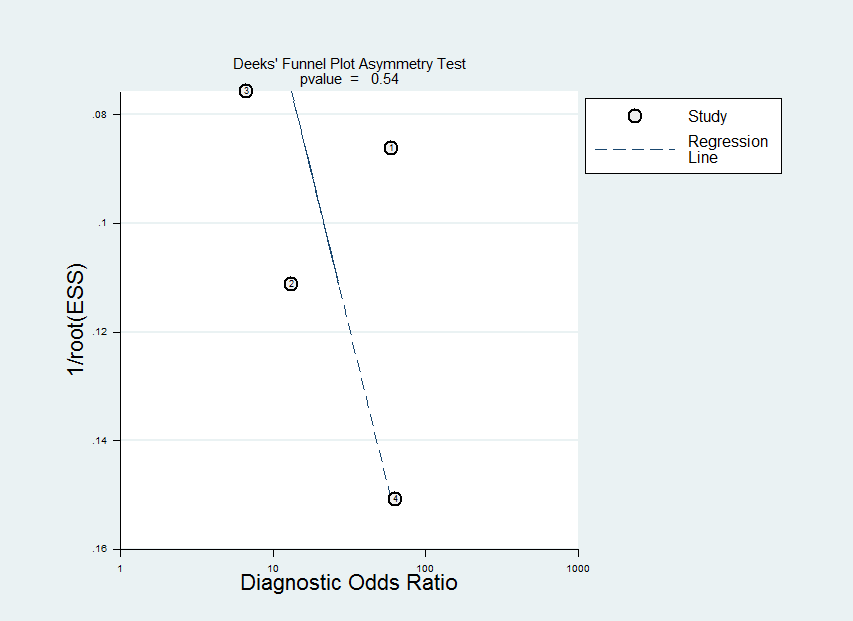


E.


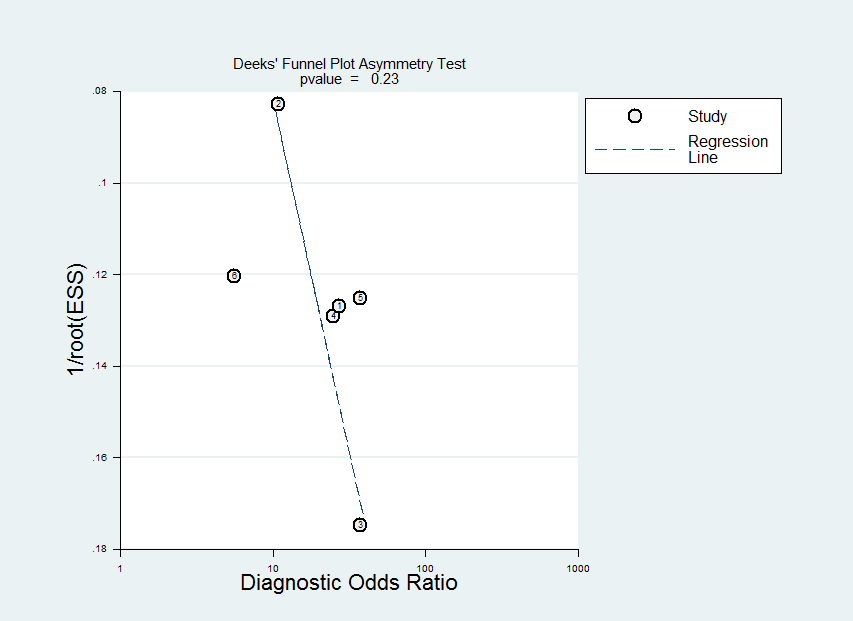


F.


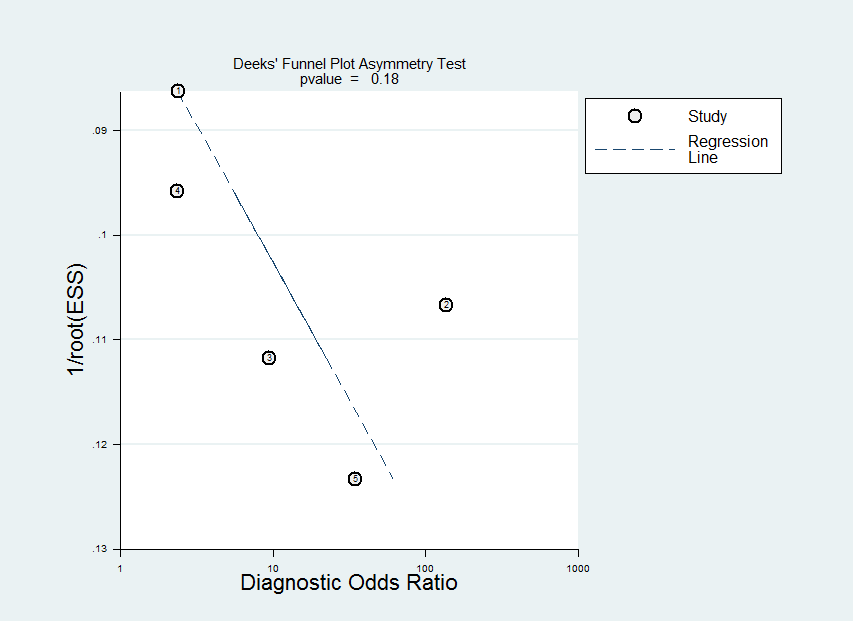


G.


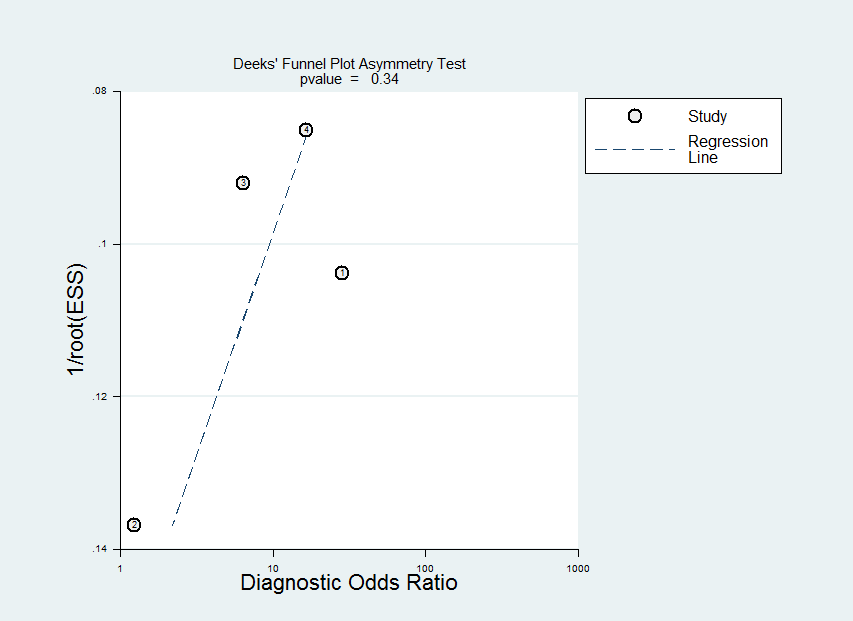

Supplement: Supplementary file 1 — Forest plot of the sensitivities and specificities reported by each interleukin. The Forest plots of the sensitivities and specificities reported by A. interleukin-6; B.interleukin-33; C interleukin-12; D interleukin-2; E interleukin-12p40. Figure S2. Funnel graph for the assessment of potential publication bias in each interleukin. The Funnel graphs for the assessment of potential publication bias in each interleukin: A for IL-27; B for IL-18;C for IL-6; D for IL-33; E for IL-12; F for IL-2; G for IL-12p40. (DOC 231 kb) [file 12890_2017_530_MOESM1_ESM.doc]
